# Discovery of Biomarker Panels for Neural Dysfunction in Inborn Errors of Amino Acid Metabolism

**DOI:** 10.1038/s41598-019-45674-2

**Published:** 2019-06-24

**Authors:** Alba-Aina Castells, Daniela Gueraldi, Rafel Balada, Alba Tristán-Noguero, Elisenda Cortès-Saladelafont, Federico Ramos, Silvia Meavilla, Mariela De Los Santos, Camila Garcia-Volpe, Roser Colomé, Maria Luz Couce, Cristina Sierra, Aida Ormazábal, Marta Batllori, Rafael Artuch, Judith Armstrong, Soledad Alcántara, Àngels Garcia-Cazorla

**Affiliations:** 1 Metabolic Unit, Departments of Neurology, Nutrition Biochemistry and Genetics, Institut Pediàtric de Recerca Sant Joan de Déu, CIBERER, ISCIII and metabERN, Hospital San Joan de Déu, Barcelona, Spain; 2 Neural Development Lab, Departament de Patologia i Terapèutica Experimental, Institut de Neurociències, Universitat de Barcelona, IDIBELL, l’Hospitalet de Llobregat, Barcelona, Spain; 3 Metabolic Unit, Department of Pediatrics, Hospital Clinico Universitario de Santiago de Compostela, Universidad de Santiago, CIBERER, IDIS, MetabERN, Santiago de Compostela, Spain

**Keywords:** Paediatric neurological disorders, Diseases of the nervous system

## Abstract

Patients with inborn errors of amino acid metabolism frequently show neuropsychiatric symptoms despite accurate metabolic control. This study aimed to gain insight into the underlying mechanisms of neural dysfunction. Here we analyzed the expression of brain-derived neurotrophic factor (BDNF) and 10 genes required for correct brain functioning in plasma and blood of patients with Urea Cycle Disorders (UCD), Maple Syrup Urine Disease (MSUD) and controls. Receiver-operating characteristic (ROC) analysis was used to evaluate sensitivity and specificity of potential biomarkers. *CACNA2D2* (α2δ2 subunit of voltage-gated calcium channels) and *MECP2* (methyl-CpG binding protein 2) mRNA and protein showed an excellent neural function biomarker signature (AUC ≥ 0,925) for recognition of MSUD. *THBS3* (thrombospondin 3) mRNA and AABA gave a very good biomarker signature (AUC 0,911) for executive-attention deficits. *THBS3*, *LIN28A* mRNA, and alanine showed a perfect biomarker signature (AUC 1) for behavioral and mood disorders. Finally, a panel of BDNF protein and at least two large neural AAs showed a perfect biomarker signature (AUC 1) for recognition of psychomotor delay, pointing to excessive protein restriction as central causative of psychomotor delay. To conclude, our study has identified promising biomarker panels for neural function evaluation, providing a base for future studies with larger samples.

## Introduction

Although inborn errors of amino acid metabolism (IEM) are treatable disorders, long-term cognitive and behavioral problems are almost constant in patients despite strict dietary management and other treatment approaches. Factors underlying brain dysfunction are not fully understood.

Urea cycle disorders (UCDs, OMIM #311250) are IEM characterized by recurrent hyperammonemic episodes, due to dysfunctions in any of the urea cycle pathway enzymes, a detoxification system that converts ammonia into urea. In the brain, astrocytes are responsible for detoxifying ammonia through glutamine synthetase (GS) and the amidation of glutamate to glutamine^1,2^. Severe enzyme defects usually manifest symptoms during the first days of life, while symptoms of partial defects tend to appear later in life. Hyperammonemia can damage cerebral tissue by altering cerebral energy metabolism, glutamine/glutamate levels, neurotransmission, and signal transduction of pathways related to neuronal survival and plasticity^3,4^. Neurocognitive dysfunctions and behavioral impairment represent common long-term outcomes that are likely to reflect chronic ammonia/glutamine toxicity^5,6^.

Maple syrup urine disease (MSUD; OMIM #248600) is caused by the deficiency of branched-chain α-keto acid dehydrogenase (BCKD) complex activity, a key enzyme for the amino acid catabolism, leading to increased levels of branched-chain amino acids (BCAAs) and their corresponding α-ketoacids^7^. The BCKD complex is composed of three catalytic components: E1, E2 and E3 and two regulatory enzymes, the BCKD kinase (BCKDK) and BCKD phosphatase (PP2Cm)^8^. Mutations in BCKDK (OMIM #614923) are associated with decreased levels of BCAAs, epilepsy, intellectual disability and autism^9^.

Patients with the most severe form of MSUD develop metabolic decompensation and encephalopathy within the first weeks of life and die if untreated^7,10,11^. Therapy is based on strict dietary management with low BCAAs (mainly leucine) content and/or liver transplantation^12,13^. Despite treatment, several studies have described small reductions in the intelligence quotient and increased rate of neuropsychiatric conditions, mainly attention deficit hyperactivity disorder (ADHD), depression and anxiety^14–18^. Neurotoxicity of BCAAs and their α-ketoacids, mainly leucine, might underlie neuropsychiatric disorders, as leucine plasma concentrations are closely correlated with acute symptoms^17^. Due to blood-brain barrier (BBB) amino acid transporter saturation, increased BCAAs and α-ketoacids in the brain, are concomitant with deficiency of essential amino acids, causing brain-specific deficits in protein synthesis, neurotransmitter depletion and inhibition of mitochondrial enzymes^8,19^.

Early detection and treatment is the gold standard to avoid long-term disabilities in IEM patients. Therefore, there is a need to find new biomarkers of neural function for the identification and management of those children that, despite metabolic management, are at risk to develop neurologic, cognitive and behavioral problems.

One currently accepted biomarker candidate for brain functioning is brain-derived neurotrophic factor (BDNF), as BDNF signaling is involved in brain development, synaptic function and plasticity^20^. Activity-dependent regulation of BDNF expression is in part mediated by transcription factor methyl-CpG binding protein 2 (MeCP2), and altered levels of MeCP2, BDNF or their downstream signaling are widely implicated in neuropsychiatric diseases and mood disorders^21,22^. Altered BDNF levels in plasma have been recently described in MSUD patients and the hippocampus of hyperammonemic rats^23,24^. BDNF is also involved in glucose and energy homeostasis through control of energy intake and expenditure^25^, in part by regulation of miRNA and protein synthesis through LIN28A RNA-binding protein^26,27^. Additional candidate biomarkers are CACNA2D1-2 genes, which codify for α2δ1-2 regulatory subunits of the voltage-gated calcium channels (VGCCs)^28^. Aside from its modulatory channel function, α2δ1-2 are essential for the formation and stabilization of new synapses by binding to oligomeric extracellular matrix glycoproteins thrombospondins (TSPs), which are codified by THBS genes^29^. L-leucine and L-isoleucine are also well-known ligands of α2δ1-2 subunits^30^ and have been postulated as necessary for their correct function^31,32^.

Therefore, to gain insight into the molecular mechanisms underlying neurological deficits in IEM patients, we analyzed in peripheral whole blood the mRNA expression of a group of genes involved in brain development and synaptic function (*ADORA2A*, *CACNA2D2*, *FMR1*, *IRAK1*, *LIN28A*, *PTEN*, *MECP2 E1/E2*, *THBS1*, *THBS3*) in a cohort patients affected by UCD, MSUD and BCKDK deficiency. Gene expression in blood was correlated with AAs profile, BDNF protein levels in plasma, and neuropsychological symptoms, in an attempt to identify specific molecular pathways altered in each disorder, as well as common mechanisms underlying the pathophysiology of the neurological impairment present in different IEMs.

## Results

### Clinical information of patients

Table 1 shows a summary of clinical data and Table 2 shows treatment details and historical biochemical parameters of IEM patients included in this study.

**Table 1:** Summary of clinical characteristics, disease onset and outcome of IEM patients.

Patient	Sex	Disorder	Defect	Age at onset	Symptoms at onset	Age at blood analysis	NPSY tests (evaluative age)	Intellectual Disability	Psychomotor delay	Executive function/ attention deficits	Other Behavioral symptoms
1	M	UCD	OTC	(^)	Asymptomatic	16 y	NO (*)	NO	NO	NO	NO
2	M	UCD	OTC	9 d	Lethargy, failure to thrive	10 y	YES (7, 11 y)	NO (IQ: 120)	NO	ADHD	Autistic traits
3	M	UCD	OTC	8 y	Seizures, learning difficulties	19 y	YES (13 y)	NO (IQ: 88)	NO	Verbal, visual and working memory deficits	Mood disorder, depression
4	F	UCD	OTC	—	Asymptomatic	36 y	NO (*)	NO	NO	NO	NO
5	M	UCD	HHH	20 m	Mild psychomotor delay, lower limb pyramidal signs	12 y	YES (7 y)	YES (IQ: 76)	YES	ADHD, inhibition and literacy affected	NO
6	M	UCD	ASL	3 y	Language delay	13 y	YES (7 y)	YES (IQ: 54)	YES	Verbal, attention and working memory deficits	Language and verbal comprehension difficulties
7	M	UCD	OTC	4 y	Cyclic vomiting hyporeactivity, seizures	13 y	YES (11 y)	NO (IQ: 106)	NO	ADHD, working memory deficits	NO
8	F	UCD	OTC	6 y	Ataxia confusion, vomit, lethargy	22 y	NO (*)	NO	NO	NO	NO
9	F	UCD	OTC	2 y	Fever, food refusal, irritability	5 y	YES (6 y)	NO (IQ:104)	NO	Attention and working memory deficits	NO
10	F	UCD	OTC	14 y	Coma	19 y	YES (16 y)	YES (IQ: 70)	YES	Attention and working memory deficits	NO
11	F	UCD	OTC	(^)	Asymptomatic	10 y	NO (*)	NO	NO	NO	NO
12	F	UCD	OTC	(+)	Asymptomatic	42 y	NO (*)	NO	NO	NO	NO
13	M	UCD	ASS	8 y	Behavior disorder, ADHD	16 y	YES (17 y)	YES (IQ: 52)	YES	ADHD, working memory deficits	Irritability, aggressiveness
14	F	UCD	OTC	8 m	Vomit, food refusal, hyporeactivity	10 y	NO	NO	NO	—	NO
15	F	UCD	OTC	3 y	Hyporeactivity	6 y	NO	NO	YES	—	NO
16	F	UCD	ASS	2 d	Lethargy	9 y	YES (12 y)	NO	YES	ADHD	Autistic traits
17	F	UCD	OTC	3 y	Lethargy, seizures, coma	9 y	YES (7 y)	YES (IQ: 50)	YES	Attention and working memory deficits	Disruptive and self-harm behavior
18	F	UCD	OTC	2 y	Coma	12 y	YES (10 y)	NO (IQ: 88)	NO	NO	NO
19	F	UCD	ASS	NS	Asymptomatic	3 y	NO (*)	NO (IQ: 107)	NO	NO	NO
20	M	MSUD	DBT (E2)	9 d	Poor feeding, lethargy	1 y	YES	NO (IQ:88)	NO	NO	NO
21	F	MSUD	BCKDHB(Ib)	10 d	Poor feeding, lethargy	19 y	YES	YES (IQ 70–85)	NO	NO	NO
22	F	MSUD	BCKDHB(Ib)	9 d	Poor feeding, lethargy	2 y	YES	NO (IQ ≥ 85)	NO	Attention deficits	NO
23	F	MSUD	DBT (E2)	11 d	Poor feeding, lethargy	3 m	YES (3 y)	NO (IQ: 93)	NO	NO	NO
24	M	MSUD	BCKDHB(Ib)	7 d	Poor feeding, coma, sweet odor smelling	13 y	YES (8 y)	NO (IQ: 89)	NO	Attention deficits	NO
25	F	MSUD	BCKDHB(Ib)	7 d	Poor feeding, lethargy, sweet odor smelling	4 m	YES (6 y)	NO (IQ:104)	NO	NO	NO
26	M	MSUD	BCKDHB(Ib)	7 d	Poor feeding, coma	19 m	YES (1 y)	NO (IQ:90)	NO	Attention and working memory deficits	NO
27	F	MSUD	BCKDHB(Ib)	NS	Asymptomatic	28 d	NO (*) (4 y)	NO (IQ:108)	NO	NO	NO
28	F	MSUD	BCKDHB(Ib)	10 d	Poor Feeding, lethargy	6 y	YES	NO (IQ ≥ 85)	NO	Attention deficits	NO
29	M	BCKDK Deficiency	BCKDK	1 m	Psychomotor delay, gastrointestinal symptoms, moderate anorexia	6 y	YES	YES (IQ < 35)	YES	YES	Autism disorder, aggressive disorder

ADHD: attention deficit hyperactivity; ASL Argininosuccinate Lyase; ASS: Argininosuccinate synthetase, HHH: Hyperornithinemia-Hyperammonemia-Homocitrullinuria, IQ: Intelligence Quotient, IQNPSY: Neuropsychological tests not done but asymptomatic and normal academic and social achievements; MSUD: Maple Syrup Urine Disease, NS: Newborn Screening; OTC: Ornithine transcarbamylase, UCD: Urea Cycle Disorders, (*)

M: male, F: female, d: days; m: months; y: years; - unknown; n.a. not applicable.

(^): diagnosed because of an affected sibling. (+): diagnosed because of an affected son.

**Table 2:** Treatment and historical biochemical data records of IEM patients.

Patients	Diagnosis	Treatment		Historical data (µmol/L)						
				N° metabolic decompensations	Am (<50)		Gln (330–754)		Leu (<200)	
					Mean	Max	Mean	Max	Mean	Max
1	OTC		L-arginine, L-citrulline Protein-restricted diet	0	25	38	585	734	—	—
2	OTC	NaPB Methylphenidate	L-arginine, L-citrulline L-carnitine Protein-restricted diet	4	53	290	865	1387	—	—
3	OTC		L-arginine, L-citrulline Protein-restricted diet	1	47	282	721	1766	—	—
4	OTC		L-citrulline, L-arginine L-carnitine Protein-restricted diet	0	31	55	727	1029	—	—
5	HHH	Methylphenidate	L-arginine, L-citrulline L-carnitine Protein-restricted diet	0	61	220	683	1060	—	—
6	ALS		L-arginine Protein-restricted diet	0	28	45	505	667	—	—
7	OTC	Methylphenidate	L-arginine, L-carnitine Protein-restricted diet	2	37	650	562	835	—	—
8	OTC		L-citrulline, L-carnitine Protein-restricted diet	1	45	247	855	1274	—	—
9	OTC	NaPB	L-arginine Protein-restricted diet	2	59	180	1203	1792	—	—
10	OTC	NaPB	L-arginine, L-citrulline Protein-restricted diet	2	30	485	800	1320	—	—
11	OTC	NaPB	L-Arginine Protein-restricted diet	0	40	67	626	796	—	—
12	OTC		L-arginine, L-citrulline Protein-restricted diet	0	36	50	797	1085	—	—
13	ASS	NaPB	L-arginine, L-carnitine Protein-restricted diet	0	34	44	804	1174	—	—
14	OTC		Liver Transplantation: Tacrolimus, Magnogene, Methylprednisolon, Septrin, Usochol, L-citrulline	1	67	669	874	1664	—	—
15	OTC	NaPB	L-citrulline Protein-restricted diet	1	51	216	723	889	—	—
16	ASS	Na-Benzoate NaPB	L-carnitine, L-arginine Protein-restricted diet	6	67	419	760	1288	—	—
17	OTC	NaPB	L-citrulline, L-carnitine Protein-restricted diet	1	—	550	—	—	—	—
18	OTC		L-citrulline, L-carnitine Protein-restricted diet	4	30	288	1000	1380	—	—
19	ASS		L-arginine	0	20	22	400	622	—	—
20	MSUD		Low BCAA diet	2	—		—	—	324	-
21	MSUD		Low BCAA diet	—	—		—	—	414	1507
22	MSUD		Low BCAA diet	5	—		—	—	278	2717
23	MSUD		Low BCAA diet	1	—		—	—	302	1760
24	MSUD		Low BCAA diet	5	—		—	—	239	1155
25	MSUD		Low BCAA diet	1	—		—	—	211	1692
26	MSUD		Low BCAA diet	1	—		—	—	162	968
27	MSUD		Low BCAA diet	0	—		—	—	95	262
28	MSUD		Low BCAA diet	5	—		—	—	378	3241
29	BCKDK		High BCAA diet	n.a.	—		—	—	101	197

Abbreviations: Am: ammonia, ASL Argininosuccinate Lyase, ASS: Argininosuccinate synthetase, Gln: glutamine, HHH: Hyperornithinemia-Hyperammonemia-Homocitrullinuria, n.a. not applicable, Leu: leucine, MSUD: Maple Syrup Urine Disease, NaPB Sodium phenylbutyrate, OTC: Ornithine transcarbamylase, UCD: Urea Cycle Disorders.

UCD patients (7 males and 12 females) include ornithine transcarbamylase deficiency (OTC deficiency) (14), citrullinemia type I (CTLN1) (3), hyperornithinemia-hyperammonemia-homocitrullinuria syndrome, (HHH syndrome) (1) and argininosuccinate lyase deficiency (ASL deficiency) (1), and five of them (26%) were asymptomatic. All UCD patients were treated with L-Arginine +/− L-Citrulline and all but two followed a protein-restricted diet (0.5–1,3 g/Kg/day). Asymptomatic patients showed mean glutamine levels <800 µmol/L; while half of symptomatic patients showed mean glutamine levels ≥800 µmol/L. Eight symptomatic patients (42%) were treated with ammonia scavengers, all with sodium phenylbutyrate (NaPB) and one also with sodium benzoate. Patients without scavengers were those with normal ammonia and amino acids in the follow-up metabolic controls. MSUD patients (3 males and 6 females) include mutations in *DBT* (2) and *BCKDHB* (6), one of them asymptomatic. We also included a patient with a genetic diagnose of BCKDK deficiency. MSUD patients followed a low BCAA diet while BCKDK deficiency patient received a high protein diet supplemented with BCAAs. Despite low BCAA diet, mean leucine levels ≥200 µmol/L (normal range in young patients <200 µmol/L) were observed in 78% of MSUD patients.

From the 29 IEM patients in our cohort, 21 symptomatic patients had a neuropsychological evaluation to detect abnormal executive functions and attention deficits (15 patients) cognitive disabilities (7 patients) and other behavioral disorders (7 patients). Three patients of those who fulfilled ADHD criteria and had poor academic results were treated with psychostimulants (methylphenidate). Neuropsychological evaluation was not performed in healthy control subjects (n = 27) and in asymptomatic patients that achieved successfully both academic and personal life events (n = 8).

### Analysis of metabolite levels

The 21 principal amino acids and 4 amino acid related-compounds were measured in all plasma samples by ion-exchange chromatography with nynhydrin detection derivatives. Ammonia levels were measured only in OTC patients. Average values for each condition are shown in Table 3.

**Table 3:** Summary of biochemical profile.

(µmol/L)	Control (n = 27)	MSUD (n = 9)	BCKDK (n = 1)	UCD (n = 19)	UCD w/sc.(n = 8)	UCD w/o sc. (n = 11)
Val	187.51 ± 34.18	201.42 ± 112,82	251.40	181.50 ± 69.15	**138.61** ± 41.30*^£^	219.62 ± 67.80
Leu	96.29 ± 19.95	**233.49** ± 158.75**	115.20	101.04 ± 43.21	72.89 ± 20.96^££^	126.06 ± 43.05
Ile	50.19 ± 9.82	**165.61** ± 103.22***	63.40	56.26 ± 25.01	41.78 ± 13.89^£^	69.14 ± 26.19
Gly	194.83 ± 44.63	**320.73** ± 104.39***	262.30	**245.21** ± 69.42*	245.03 ± 67.71	245.37 ± 75.02
Ala	305.92 ± 57.86	311.02 ± 134.13	492.00	365.24 ± 148.94	407.30 ± 179.26	327.86 ± 113.61
Ser	110.16 ± 24.56	**146.30** ± 34.32**	169.80	116.05 ± 30.19	108.49 ± 21.24	122.77 ± 36.34
Pro	147.81 ± 39.78	208.28 ± 87.01	273.30	157.75 ± 43.21	151.69 ± 49.32	163.19 ± 39.19
Thr	111.58 ± 22.07	**204.50** ± 92.04**^##^	189.10	100.32 ± 42.40	83.89 ± 34.41	114.92 ± 45.26
Cys	31.13 ± 5.81	29.31 ± 7.59	35.60	33.72 ± 7.72	30.59 ± 7.78	36.51 ± 6.91
Met	19.05 ± 3.46	22.94 ± 6.29	49.10	**26.80** ± 7.66***	26.86 ± 9.66	26.74 ± 5.97**
Phe	50.23 ± 9.05	61.60 ± 22.50	95.00	46.64 ± 11.98	**39.25** ± 7.41*^£^	53.14 ± 11.69
Tyr	57.55 ± 15.65	76.21 ± 32.77	140.40	55.33 ± 17.41	50.55 ± 19.88	59.58 ± 14.73
Trp	52.78 ± 11.66	69.26 ± 25.81	99.80	53.61 ± 19.15	45.65 ± 20.21	60.68 ± 16.02
Asp	5.39 ± 3.36	6.31 ± 2.30	4.30	4.86 ± 2.77	4.36 ± 1.95	5.30 ± 3.40
Glu	25.96 ± 20.88	**39.16** ± 18.79*	22.20	28.36 ± 10.74	34.39 ± 8.73	23.00 ± 9.76
Gln	414.87 ± 50.12	478.48 ± 92.18^#^	579.00	**666.14** ± 146.69***	**753.53** ± 138.38***	**588.46** ± 108.89**
Asn	52.78 ± 8.51	**44.27** ± 15.94*^##^	98.80	**64.64** ± 19.80*	58.66 ± 6.54	69.94 ± 26.06
Hys	71.27 ± 9.95	**81.39** ± 10.83*	110.10	**90.16** ± 20.03***	**84.83** ± 8.10*	**94.90** ± 26.29*
Lys	142.68 ± 16.61	183.19 ± 46.93	281.00	143.41 ± 49.44	127.75 ± 26.96	157.33 ± 61.56
Arg	54.61 ± 13.84	67.67 ± 34.43	90.90	66.33 ± 27.41	65.50 ± 28.66	67.07 ± 27.99
Orn	64.11 ± 21.45	**90.80** ± 31.68*	72.80	97.83 ± 90.00	75.81 ± 36.94	117.40 ± 118.70
Tau	47.82 ± 21.75	**93.97** ± 38.38***^##^	39.10	42.57 ± 19.56	45.76 ± 27.97	39.73 ± 7.82
Cit	27.16 ± 7.62	28.28 ± 4.65	28.70	258.55 ± 657.39	514.23 ± 919.91	31.28 ± 19.42
Oh-Pro	12.73 ± 6.11	15.98 ± 6.92	16.50	15.48 ± 8.94	12.25 ± 4.35	18.34 ± 11.13
AABA	20.70 ± 6.09	20.64 ± 4.06	4.50	22.76 ± 11.15	19.29 ± 6.48	25.84 ± 13.75
Ammonia	—	—	—	35.94 ± 22.38	45.33 ± 33.03	30.30 ± 11.61

Results are presented as mean ± standard deviation. *Show statistical differences with respect to control, ^#^with respect to UCD and ^£^with respect to UCD without ammonia scavengers and assessed by Kruskal Wallis test. ^*/£/#^P < 0.05, **^/££/##^p < 0.01, ***^/£££/###^p < 0.001. w/sc. with scavenger treatment, w/o sc. without scavenger treatment.

Despite metabolic management, several mean AAs values were out of normal range both in UCD and MSUD groups (Table 3). The mean ammonia level in the UCD group was in the normal range but nitrogen-rich AAs glutamine (p > 0.0001) and asparagine (p = 0.014) were significantly increased in UCD patients with respect to controls; and as were with glycine (p = 0.014), methionine (p > 0.0001) and histidine (p = 0.000). As expected, citrulline was only increased in patients with ASS deficiency (p = 0.04). When segregating UCD patients by treatment, patients treated with ammonia scavengers showed specific downregulations of phenylalanine (p = 0.045) and BCAAs (Val p = 0.013; Leu p = 0.003; Ile, p = 0.015) with respect to untreated UCD patients and phenylalanine (p = 0.032) and valine (p = 0.041) also with respect to controls.

BCAAs levels were high in most MSUD patients with leucine (p = 0.01) and isoleucine (p = 0.000) over the control group. glycine (p = 0.000), taurine (p = 0.000), serine (p = 0.004), threonine (p = 0.002), glutamate (p = 0.024), asparagine (p = 0.019), histidine (p = 0.017) and ornithine (p = 0.026) were also significantly elevated in MSUD group with respect to controls. The only BCKDK deficiency patient analyzed showed normal BCAAs, a marked decrease in α-Aminobutyric acid (AABA) and levels above the normal range in other twelve AAs (Table 3).

### mRNA and protein expression analysis

To ensure the reliability of RT-PCR analysis we set strict sample exclusion criteria (see patients and methods) and as a result, 27 controls, 16 UCD, 7 MSUD, and 1 BCKDK deficiency patients were included in mRNA and further analysis. In addition, outliers were also excluded from the analysis.

A summary of significant differences in mRNA RT-PCR analysis is shown in Table 4. MSUD and UCD groups exhibit significant differences in mRNA gene expression profiles with respect to controls and between them. All genes analyzed in the BCKDK deficiency patient were in the control range. In MSUD patients, *CACNA2D2*, and *MECP2 E1* and *E2* isoforms mRNAs were significantly downregulated (p = 0.007; p = 0.027; p = 0.001 respectively), while *THBS1* mRNA was upregulated (p = 0.047) with respect to controls. In UCD patients, *THBS1* and *LIN28A* mRNA were upregulated with respect to controls (p = 0.031; p = 0.023 respectively). No differences between groups were found for *FMR1*, *IRAK1*, *PTEN*, and *THBS3*.

**Table 4:** Gene and BDNF protein expression in peripheral blood.

		Control (n = 28)	MSUD (n = 7)	BCKDK (n = 1)	UCD (n = 16)	UCD w/sc. (n = 8)	UCD w/o sc. (n = 8)
RQ	*ADORA2A*	1.03 ± 0.24	1.13 ± 0.21	1.304	1.11 ± 0.35	1.30 ± 0.35*^£^	0.93 ± 0.25
	*CACNA2D2*	1.03 ± 0.34	**0.55** ± 0.20**^##^	0.884	1.03 ± 0.22	1.08 ± 0.24	0.96 ± 0.19
	*FMR1*	1.05 ± 035	0.99 ± 0.12	—	1.21 ± 0.21	1.18 ± 0.18	1.23 ± 0.25
	*IRAK1*	1.02 ± 0.17	1.07 ± 0.17	1.218	1.10 ± 0.24	1.14 ± 0.24	1.06 ± 0.26
	*LIN28A*	1.12 ± 0.72	0.65 ± 0.20^##^	0.831	**2.42** ± 1.92*	1.82 ± 1.11	3.03 ± 2.42
	*MECP2 E1*	1.00 ± 0.23	**0.79** ± 0.17*^#^	—	1.15 ± 0.28	**1.23** ± 0.28*	1.04 ± 0.26
	*MECP2 E2*	1.05 ± 0.14	**0.79** ± 0.08***^###^	1.150	1.01 ± 0.18	0.99 ± 0.20	1.02 ± 0.16
	*PTEN*	1.04 ± 0.31	0.98 ± 0.47	0.826	1.42 ± 0.61	1.24 ± 0.46	1.60 ± 0.72
	*THBS1*	0.99 ± 0.56	**1.73** ± 0.66*	0.713	**1.54** ± 0.81*	**1.80** ± 0.95*	1.29 ± 0.58
	*THBS3*	1.00 ± 0.21	1.01 ± 0.06	1.140	1.14 ± 0.22	1.16 ± 0.23	1.11 ± 0.22
ng/mL	BDNF	136.20 ± 88.54	122.68 ± 68.03	—	77.64 ± 61.46	93.22 ± 85.24	67.26 ± 42.01

Data are shown as mean ± standard deviation. *Show statistical differences with respect to control, ^#^with respect to UCD and ^£^with respect to UCD without ammonia scavengers assessed by Kruskal Wallis test. *^/£/#^P < 0.05. **^/££/##^p < 0.01. ***^/£££/###^p < 0.001. w/sc. with scavenger treatment. w/o sc. without scavenger treatment.

When UCD patients were segregated by ammonia scavenger treatment, *ADORA2A* mRNA was significantly increased in those treated with scavengers (n = 8) with respect to control group (p = 0.042) and UCD untreated group (n = 8, p = 0.021). In addition, *MECP2 E1* (p = 0.047) and *THBS1* (p = 0.031), mRNA were significantly increased in those treated with scavengers with respect to control group, but not to untreated UCD patients. Taken together these results suggest that ammonia scavenger treatment might directly increase, *THBS1*, *MECP2 E1*, and *ADORA2A* mRNA expression.

**Figure 1:**
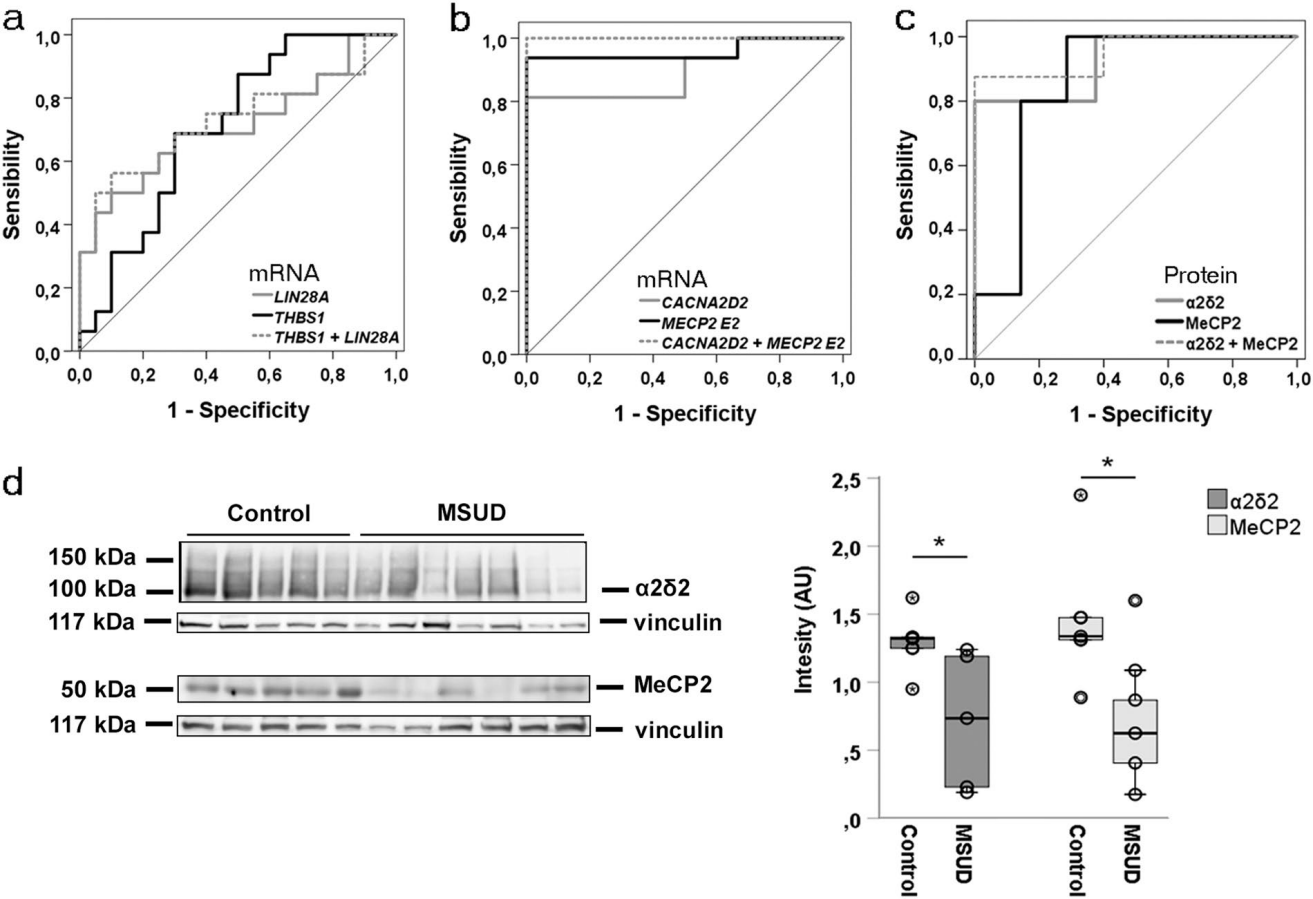
ROC curves of different neural function biomarker combinations for diagnosis between MSUD or UCD patients and healthy controls. (**a)** UCD; (**b**) MSUD mRNA; (**c**) MSUD protein; (**d**) Representative western blot and densitometric analysis showing significant differences for α2δ2 and MeCP2 in MSUD patients (n = 8) and controls (n = 5). *p < 0.05, Mann-Whitney U test.

To evaluate the protein expression of the main potential gene biomarkers identified in this study, we performed western blot analysis in purified leukocytes from the same blood samples. We found that protein levels of α2δ2 and MeCP2 in leukocytes from MSUD patients (n = 8) was significantly lower than in controls (n = 5) (p = 0.011, p = 0.048, respectively), corroborating our mRNA results in whole blood (Fig. 1d). Finally, BDNF protein was measured by ELISA in plasma from the same samples included in the mRNA analysis. BDNF levels were highly variable, even in controls, and although no statistically significant differences were found between groups, there was a tendency of lower levels of BDNF in UCD patients (p = 0.061).

### Spearman correlation between levels of candidate biomarkers and amino acids

**Figure 2:**
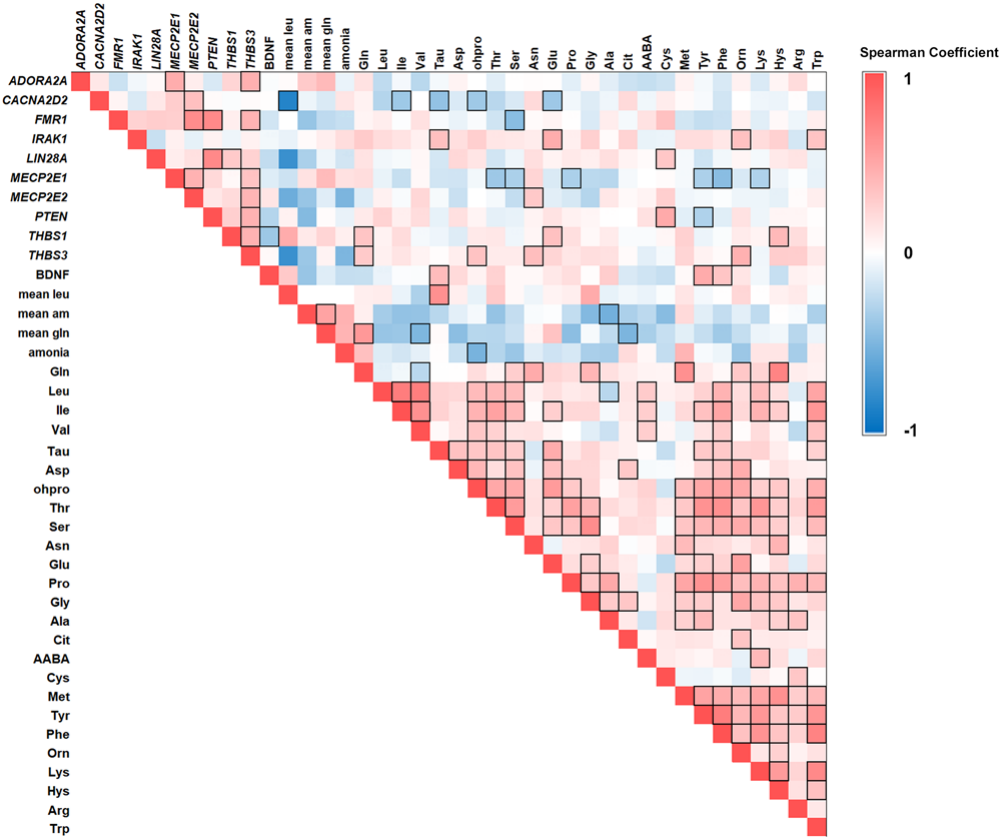
Correlations matrix heat maps. Heat map of Spearman correlation between amino acids and potential gene biomarkers performed between each biomarker for the overall population analyzed. 1 is positive correlation. 0 no correlation and −1 is negative correlation. Statistically significant correlations (p < 0.05) are black boxed.

We then investigated correlations among potential neural function biomarkers and relevant amino acids using Spearman coefficient of pairwise comparison between samples (Fig. 2). Correlations were performed between each candidate biomarker for the overall population and relevant statistically significant correlations were identified from the correlation matrix heat map obtained. Spearman coefficient (ρ) of 1 or −1 represents perfect positive or negative correlation respectively.

We found a positive correlation between glutamine levels in plasma and mRNA levels in blood of *THBS1* (ρ = 0.342; p = 0.025) and *THBS3* (ρ = 0.313; p = 0.041), which were also positively correlated between them (ρ = 0.449; p = 0.005). In addition, *THBS1* mRNA positively correlated with *LIN28A* mRNA (ρ = 0.312; p = 0.047), and negatively with BDNF protein levels (ρ = −0.348; p = 0.019); while *THBS3* mRNA positively correlated with mRNA levels of *MECP2 e1* and *e2* isoforms (ρ = 0.353/0.433; p = 0.032/0.007 respectively), *PTEN* (ρ = 0.450; p = 0.006) and *FMR1* (ρ = 0.447; p = 0.033). We also found a significant strong negative correlation between mean historical leucine levels and BCAAs receptor *CACNA2D2* mRNA (ρ = −0.857; p = 0.014); and a significant positive correlation between mean historical leucine levels and taurine (ρ = 0.648; p = 0.043). Consistently, *CACNA2D2* mRNA also had a significant negative correlation with taurine (ρ = −0.420; p = 0.006), isoleucine (ρ = −0.377; p = 0.015) and glutamate (ρ = −0.378; p = 0.015), three AAs that are significantly increased in MSUD patients.

### Identification of UCD and MSUD gene biomarker signature

To validate the potential diagnostic and recognition effectiveness of neural function biomarker panels, ROC analysis was applied and the area under the ROC curve (AUC) was calculated for each candidate biomarker. AUC value varies from 0 to 1, where values 0.8 > AUC < 0.9 and 0.9 > AUC < 1 reflect good and very good biomarker performance respectively.

We first tested sensibility and specificity of candidate biomarkers altered in MSUD or UCD patients with respect to control group, and a summary of relevant potential gene biomarkers is shown in Table 5 and Fig. 1. From BDNF and the 10 genes analyzed in this study only *LIN28A* (AUC = 0.712) and *THBS1* (AUC = 0.707) exhibit fair biomarker accuracy for UCD. On the contrary, in the case of MSUD, good or very good biomarker performance was found for *MECP2 (*AUC *E2* mRNA = 0.942, *E1* mRNA = 0.802, MeCP2 protein = 0.857), *CACNA2D2* (AUC *CACNA2D2* mRNA = 0.865, α2δ2 protein = 0.925) and with less accuracy *THBS1* (AUC mRNA = 0.791). Biomarker specificity and sensitivity are maximal when *MECP2 E2* and *CACNA2D2* mRNA are simultaneously considered (AUC mRNA = 1, protein = 0.95). Taking together, these results suggest that altered α2δ2 and MeCP2 signaling might be involved in the neural function deficits present in MSUD patients.

**Table 5:** Area under the curves (AUC) of the biomarker combinations for MSUD and UCD disorders calculated with respect to healthy control group.

Biomarker Panel	MSUD				UCD			
	AUC	Desv error	p value	Trend	AUC	Desv error	p value	Trend
Leu	0.786	0.111	0.011	↑				
Ile	0.901	0.082	<0.001	↑				
Tau	0.889	0.055	0.001	↑				
Glu	0.753	0.085	0.025	↑				
Gly	0.909	0.048	<0.001	↑				
Gln					0.972	0.027	<0.001	↑
*CACNA2D2*	0.865	0.720	0.007	↓				
*LIN28A*	0.716	0.092	0.133	↓	0.712	0.088	0.023	↑
*MECP2 E1*	0.802	0.090	0.027	↓				
*MECP2 E2*	0.942	0.045	0.001	↓				
*THBS1*	0.791	0.089	0.046	↑	0.707	0.083	0.031	↑
*MECP2 E1* + *MECP2 E2*	0.954	0.042	0.001					
*MECP2 E2* + *CACNA2D2*	1.000	<0.001	<0.001					
*THBS1* + *CACNA2D2*	0.953	0.044	0.003					
*THBS1* + *LIN28A*					0.731	0.090	0.018	
α2δ2	0.925	0.081	0.013	↓				
MeCP2	0.857	0.112	0.042	↓				
α2δ2 + MeCP2	0.95	0.06	0.008					

### Identification of gene biomarker signature of neural function in IEM patients

We used the Kruskal Wallis test to identify significant correlations between candidate biomarkers and the presence of neuropsychological symptoms. No significant correlations were found between individual candidate mRNA biomarkers or BDNF and neuropsychological symptoms within MSUD or UCD patients, and the only BCKDK deficiency patient included in the study precludes this type of analysis.

**Figure 3:**
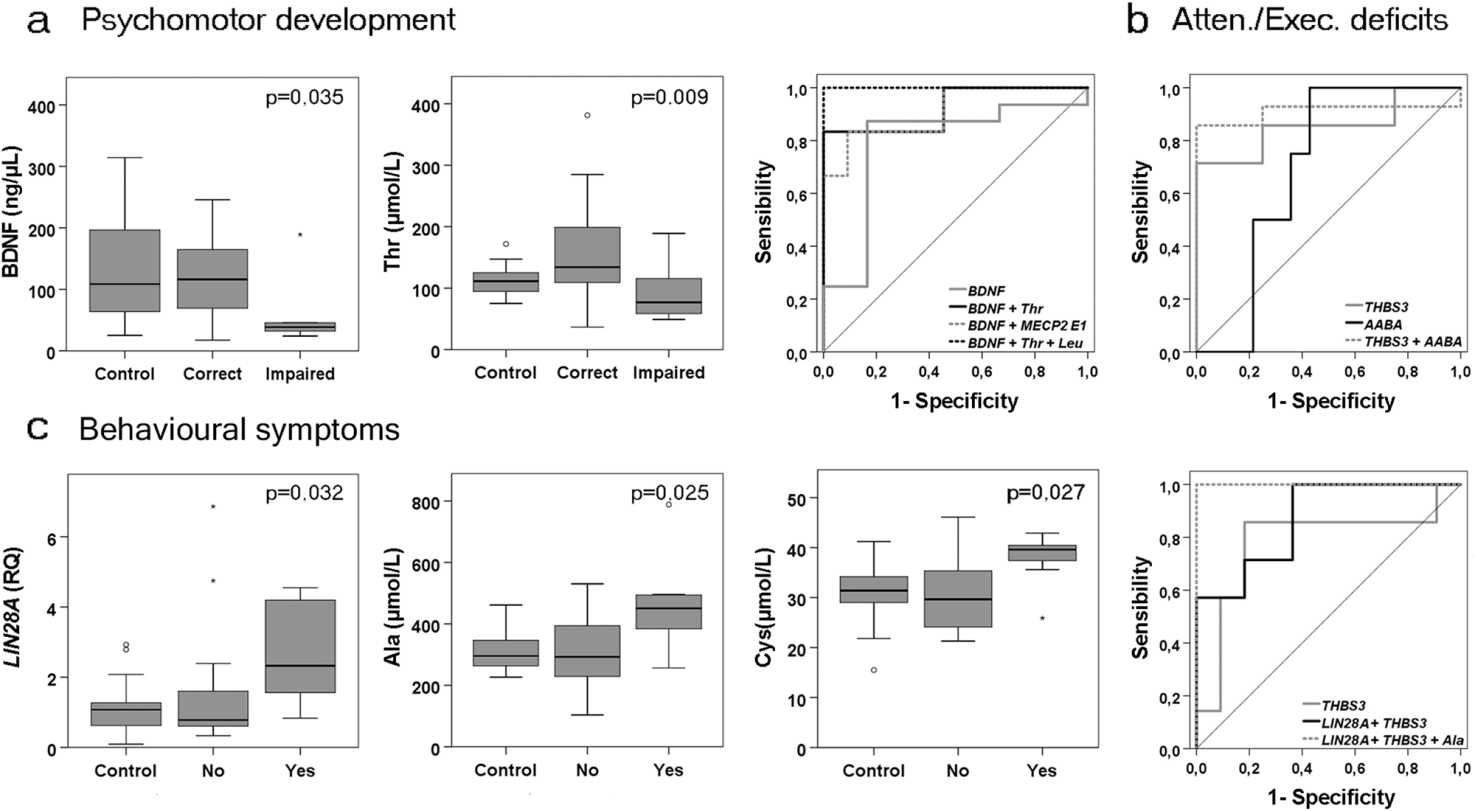
Correlation graphs and ROC curves for candidate biomarkers of neural dysfunction in IEM patients. (**a**) Graphs showing changes in BDNF and threonine in plasma of patients with deficits in psychomotor development and the ROC curves for different biomarker combinations calculated comparing affected with non-affected IEM patients. (**b**) ROC curves for *THBS3* mRNA in blood and AABA in plasma and its combination for the detection of deficits in attention and/or executive functions calculated comparing affected with non-affected IEM patients. (**c**) Graphs showing changes in *LIN28A* mRNA in blood and alanine and cysteine in plasma of patients with other behavioral symptoms and the ROC curves for *LIN28A*. *THBS3* and alanine biomarker combinations calculated comparing affected with non-affected IEM patients.

To identify common traits for neuropsychological deficits in IEMs we then analyzed all patient samples as a whole. Low BDNF and threonine levels in plasma, a clinical indicator of protein restriction, have a statistically significant correlation with impaired psychomotor development (BDNF p = 0.035; Thr p = 0.009) (Fig. 3a). On the other hand, increased *LIN28A* mRNA expression in blood and increased levels of alanine and cysteine in plasma significantly correlated with the presence of other behavioral symptoms (*LIN28A* p = 0.032; Ala p = 0.025; Cys p = 0.027) (Fig. 3c). Nevertheless, we could not detect LIN28A protein expression in purified leukocytes samples.

We then calculated AUC coefficients and a summary of the relevant potential effectiveness of candidate neural function biomarkers is shown in Table 6. For psychomotor delay, the combination of BDNF in plasma and *MECP2 E1* mRNA in whole blood exhibited very good biomarker accuracy (AUC = 0.909). However, accuracy improved when BDNF was combined with serine or threonine in plasma, reaching maximal accuracy (AUC = 1) when a third amino acid between leucine, isoleucine or citrulline was added (Fig. 3a). Unexpectedly, although no significant differences were found in mRNA in whole blood or in protein expression in leukocytes (data not shown), *THBS3* mRNA exhibited good biomarker performance for executive functions and attention deficits (AUC = 0.857). Biomarker performance increased when *THBS3* was combined with AABA in plasma (AUC = 0.911) (Fig. 3b). Finally, the combination of *THBS3* and *LIN28A* mRNA exhibited good biomarker performance to detect other behavioral symptoms (AUC = 0.870) in IEM patients, reaching maximal accuracy when was added alanine levels in plasma (AUC = 1) (Fig. 3c).

**Table 6:** Area under the curves (AUC) of the biomarker combinations for neuropsychiatric function in IEM patients calculated comparing affected with non-affected patients.

Biomarker Panel	Psychomotor Delay				Executive/Attention Deficits				Other Behavioral Symptoms			
	AUC	Desv error	p value	Trend	AUC	Desv error	p value	Trend	AUC	Desv error	p value	Trend
Leu	0.796	0.086	0.017	↓	0.753	0.098	0.035	↓				
Ile	0.803	0.085	0.015	↓								
Ala									0.793	0.101	0.023	↑
Ser	0.757	0.110	0.038	↓								
Thr	0.776	0.104	0.026	↓								
Cys									0.779	0.098	0.031	↑
Phe	0.763	0.116	0.034	↓								
Lys	0.743	0.019	0.049	↓								
Cit	0.809	0.110	0.013	↑					0.754	0.137	0.050	↑
AABA	0.743	0.113	0.049	↓	0.777	0.096	0.021	↓				
*LIN28A*									0.771	0.101	0.045	↑
*MECP2 E1*	0.780	0.125	0.043	↑								
*MECP2 E2*	0.733	0.127	0.084	↑					0.762	0.124	0.053	↑
*THBS3*					0.857	0.091	0.034	↑	0.791	0.127	0.036	↑
BDNF	0.792	0.120	0.039	↓								
BDNF + Ser	0.964	0.041	0.001									
BDNF + Thr	0.940	0.062	0.002									
BDNF + MECP2 E1	0.909	0.080	0.007									
BDNF + (Thr/Ser/Phe) + (Ile/Leu)	1.000	>0.000	0.001									
BDNF + (Thr/Ser) + Cit	1.000	>0.000	0.001									
*THBS3* + AABA					0.911	0.073	0.015					
*LIN28A* + *THBS3*									0.870	0.085	0.010	
*LIN28A* + *THBS3* + Ala									1.000	>0.000	>0.000	

Taking together, these data point to excessive protein restriction and reduced BDNF levels as hallmarks of psychomotor delay in IEM patients, and point to *THBS3* signaling as involved in executive and attention functions, and *THBS3* with *LIN28A* in behavior control.

## Discussion

UCD and MSUD are intoxication-type IEM while BCKDK deficiency results in BCAAs deficits, and despite correct metabolic management, affected patients frequently suffer psychomotor delay, cognitive, behavioral and psychiatric conditions. To date, early detection and rigorous medical follow-up is the gold standard to minimize neuropsychiatric sequelae^19,33,34^.

Several studies have assessed amino acid expression in plasma samples of UCD and MSUD patients. However, no biomarkers directly correlated with the neuropsychological features, and the molecular mechanisms involved are still poorly understood^35,36^. To our knowledge, this is the first study where the mRNA expression of genes involved in brain development and synaptic function were assessed in blood from IEM patients and correlated with BDNF and metabolite profiles in plasma.

The main limitation of this study is the reduced sample size (UCD n = 19, MSUD n = 9, and BCKDK deficiency n = 1), as IEMs are rare disorders. The use of blood is also challenging, as biomarker levels in blood can be highly variable and do not directly correlate with levels in brain, especially in diseases with preserved BBB integrity as IEMs. Even with these limitations, we found specific biomarker signatures for MSUD patients and neuropsychiatric dysfunctions in IEMs patients, that pointed to *CACNA2D2*, *THBS1*,*3*, *MECP2*, and *LIN28A* as new potential phenotype modifiers, after further investigation and verification with larger samples.

The first conclusion of this study is that despite metabolic management, most of symptomatic UCD and MSUD patients in our cohort still had glutamine and leucine/isoleucine respectively in the upper limits or above the normal range, and general AAs plasma profile in patients significantly differed from healthy controls.

In the case of MSUD, our data corroborate previous studies conducted in Brazilian and Filipino patients affected with MSUD which reported altered biochemical profile but no correlation between biochemical markers in serum and neuropsychological features^23,35^. However, we did not find a significant reduction in plasma BDNF levels as reported in Brazilian patients^23^, which might reflect different metabolic management and nutritional status in the two MSUD cohorts. In our cohort, none of the MSUD patients reported psychomotor delay while it was highly prevalent in the Brazilian cohort (40%).

BDNF levels are highly variable and directly related to food intake and energy metabolism^37^ and a reduction in BDNF expression was found in brains of rat offspring after maternal low-protein diet^38^. In this sense, 42% of our IEM cohort presented some degree of psychomotor delay, mostly corresponding to UCD patients treated with sodium phenylbutyrate, an ammonia scavenger known to produce a selective reduction in BCAAs^39^. We have found a significant inverse correlation between psychomotor delay and levels in plasma of BDNF and essential neutral AAs (mainly threonine, serine, phenylalanine, and BCAAs). These results highlight the necessity of careful metabolic surveillance and diet formulation based on the changing energy requirements of growing patients, as excessive essential neutral amino acid restriction and low BDNF seems to be a hallmark of psychomotor delay.

Nevertheless, we cannot rule out a role for altered BDNF in MSUD neurocognitive and behavioral sequelae, as we have found statistically significant reductions in the expression of *MECP2* mRNA, an epigenetic regulator of BDNF transcription^40,41^. In addition, we have found statistically significant reductions in *CACNA2D2* mRNA in MSUD patients, which was inversely correlated with chronically elevated BCAAs levels. We also found increased levels of *THBS1* in MSUD and UCD patients, which expression was unrelated to BCAAs levels but correlated positively with glutamine and negatively with BDNF plasma levels. Results that are in accordance with published reports showing TSP1 upregulation in mice brains with decreased levels of BDNF^42^. TSP1, the protein codified by *THBS1*, is an extracellular matrix (ECM) glycoprotein with antiangiogenic properties that exerts multifunctional effects by binding cell-surface receptors and other proteins in the ECM. TSP1 is up-regulated with injury, chronic pathologies and in response to glucose and AAs, being considered a potent modulator of human diseases^43–45^.

One of the most important findings of this work is the identification, at the level of mRNA and protein, of a biomarker signature specific at for MSUD that includes α2δ2 (AUC 0.865/0.925) and MeCP2 (AUC 0.942/0.857), as the combination of both yielded the excellent biomarker accuracy (AUC 1/0.95). No specific biomarker signature was found for UCD.

VGCCs auxiliary subunits α2δ1-2, codified by *CACNA2D1-2* genes, regulate calcium ion entry in response to electrical activity in excitable cells^46,47^. In neurons, α2δ1-2 expression modulates neurotransmitter release probability, trafficking and gating properties of AMPA-selective glutamate receptors (AMPARs)^48,49^, and have been involved in learning, memory, anxiety-related behaviors and epilepsy in rats and humans^50–54^. α2δ1-2 subunits are also essential for the formation and stabilization of new synapses by binding to TSP1-4 secreted by astrocytes^29,55^. It is assumed that BCAAs can induce conformational changes that indirectly affect TSPs binding to α2δ1-2 [29, 30, 31]. Moreover, α2δ subunits are the main targets of gabapentinoid drugs (gabapentin and pregabalin), which compete for the same binding site than BCAAs, blocking α2δ synaptogenic functions^29^, and biochemically mimicking the action of BCAAs^56^.

MeCP2 is an epigenetic regulator that preferentially binds to methylated CpG sites in promoter regions of DNA and is crucial for the correct brain development and the stability control of the neural network in response to activity^57–59^. Excessive or defective MECP2 function causes neurodevelopmental disorders associated with mental retardation, epilepsy, loss of speech and anxiety, among others^60–65^.

In addition to the above described, glycine and taurine were significantly increased in MSUD patients, data that is coincidental with previous studies^23,35^, and they stand out for its very good MSUD biomarker performance (AUC glycine 0.909 and AUC taurine 0.889). Glycine and taurine are abundant in brain, where they are involved in several aspects of normal development including neurogenesis, neuronal migration and differentiation^66,67^. Both AAs act as neurotransmitters by binding to glycine receptors, to NMDA receptors (glycine) and to GABA receptors (taurine). In addition, taurine inhibits K^+^-Cl^−^ cotransporter KCC2, modulating Cl^−^ homeostasis, the functionality of inhibitory neurotransmission and neuronal excitability^68,69^. Thus, the increase in glycine and taurine together with the reduced levels of α2δ2 and MeCP2 observed in MSUD patients might reflect an altered synaptogenesis and misbalanced excitatory-inhibitory neural network.

Unexpectedly, although no significant differences were found, *THBS3* mRNA together with AABA in plasma has revealed a good biomarker performance for executive function, attention deficits (AUC 0,911). Reduced AABA in plasma is a frequent consequence of low protein diets and it has been recently associated with depression in older Japanese patients^70^. TSP3 is developmentally regulated^44^ and few is known about functions. It has been involved in modulating integrin membrane expression and function^71^, which is also essential for the formation of correct synaptic structures^72^. As TSP3 is a secreted protein, ELISA analysis in plasma would be required to gain insight into their potential biomarker significance.

Finally, increased levels of *LIN28A* mRNA, alanine, and cysteine correlated with behavioral dysfunctions, which include autism spectrum disorder, depression or aggressiveness, and the combination of *THBS3* with *LIN28A* and alanine gave a perfect biomarker signature (AUC 1). LIN28 RNA binding proteins inhibit the biogenesis of let-7 family of miRNAs, a group of miRNAs crucial for embryonic and postnatal development, being at least the 50% of miRNAs present in mature neurons^73,74^. LIN28/let-7 pathway has been involved in controlling cell growth and energy metabolism^26^, and regulate dendritic growth and cell survival in response to BDNF^75^. Elevated levels of LIN28 and disruption of let-7 biogenesis have been described in animal models and in blood of patients with major depression^76,77^. Previous studies described that elevated levels of cysteine in infants derived in behavioral deficits in adults^78^ and plasma levels of alanine have been proposed as a marker of depression severity^79^. Taking together, our results are in concordance of what is observed in depression, which is commonly described in patients with inborn errors of metabolism, including UCD and MSUD^80,81^.

Our study, though preliminary, has identified several potential biomarker panels for neural function evaluation, providing a base for future studies. Most importantly, α2δ2 and MeCP2 showed an excellent neural function biomarker signature for MSUD. In addition, *THBS3* mRNA and AABA gave a very good biomarker signature for executive and attention deficits. *THBS3* and *LIN28A* mRNA and alanine showed a perfect biomarker signature for behavioral and mood disorders. Finally, a panel of BDNF and large neutral AAs showed a perfect biomarker signature for psychomotor delay, pointing to excessive protein restriction as potential causatives of psychomotor delay in diet-treated IEM patients. Although these results are very promising, a large number of clinical samples should be collected and the potential biomarkers in each panel quantified at the protein level for the ultimate goal to translate these neural function biomarkers to the clinical practice.

## Material and Methods

### Ethical statement

Research Ethics Committee of both hospitals, Sant Joan de Déu and Santiago de Compostela Hospitals, approved the study and informed consent was subscribed by patients and controls (when >18 years old) or by their parents (when <18 years old) prior to the collection of data. All methods were performed in accordance with the relevant guidelines and regulations.

### Subjects and assessment

19 patients with a diagnosis of UCDs, 9 patients diagnosed with MSUD, 1 patient with BCKDK (branched-chain ketoacid dehydrogenase kinase deficiency) and 27 healthy age-matched control subjects were recruited for the study at Sant Joan de Déu Hospital in Barcelona and University Hospital of Santiago de Compostela, Spain. Patients were followed in their respective center with the same standardized protocol (Haeberle *et al*. 2012) from the date of diagnosis up to current date. Controls were healthy age-matched individuals with no history of learning difficulties, psychiatric and behavioral problems, who underwent blood analysis in the context of minor surgical interventions.

Inclusion criteria were patients with genetic and/or enzymatic diagnosis of UCDs, MSUD, and BCKDK; asymptomatic and female carriers of OTC deficiency were also included because of abnormal metabolic profile that required a protein-restricted diet. Table 1 summarizes the clinical characteristics of the patients. We evaluated the following parameters: age of symptoms onset, psychomotor development and number of decompensations (defined as symptomatic hyperammonemia episode with plasma ammonia greater than 100 μmol/L or amino acid decompensation with leucine greater than 1000 µmol/L). Cognitive functions were assessed by Wechsler Intelligence Scale for Children (WISC)-IV or Kaufman Brief Intelligence Test, Second Edition (K-BIT), considering five severity levels for intellectual disability: Borderline = IQ 70–85, Mild = IQ 55–70; Moderate = IQ 40–55; Severe IQ 25–40; Profound IQ < 25. Behavioral disorders, attention, and executive functions were assessed by NEPSY-II subtests, Behavior Rating Inventory for Executive Functions (BRIEF) and Conners’ Continuous Performance Test II (CPT II). ADHD rating scale –IV (Dupaul) was used for behavioral characterization associated with attention-deficit hyperactivity disorder.

### Peripheral blood sample collection

Venous blood samples were collected in an anticoagulation EDTA tube after overnight fasting. Biochemical measurements of plasma ammonia, and amino acids were assessed by spectrophotometric technique and ion-exchange chromatography respectively.

### Gene expression assays

RNA from 500 µL of whole blood was extracted using miRCURY™ RNA Isolation Kit - Cell & Plant (300110 Exiqon) following supplier instructions. RNA concentration and purity were analyzed in IMPLEN NanoPhotometer® P-Class (Implen). RNA samples (0.5–1 µg) were reverse transcribed to cDNA (High Capacity cDNA Reverse Transcription Kit, 4368814, Applied Biosystems) and Real-Time PCR was performed using TaqMan PCR Assays (4331182, Applied Biosystems) with TaqMan Universal PCR Master Mix (4324018, Applied Biosystems) in the 7900HT Real-Time PCR System (Applied Biosystems). Assays analyzed were: *ADORA2A* (Hs00169123_m1), *CACNA2D2* (Hs01021049_m1), *FMR1* (Hs00924547_m1), *IRAK1* (Hs01018347_m1), *LIN28A* (Hs00702808_s1), *PTEN* (Hs02621230_s1), *MECP2 E1* (Hs01598237_m1), *MECP2 E2* (Hs00172845_m1), *THBS1* (Hs00962908_m1), *THBS3* (Hs00938498_m1). *GUSB* (Hs00939627_m1) and *GAPDH* (Hs99999905_m1) were used as endogenous controls. Data analysis was performed with Expression Suite Software (Life Technologies) and data expressed as Relative Quantification (RQ) normalized with respect to gusb. Sample exclusion criteria were: threshold cycles (Cts) of more than 35, replicates with a standard deviation greater than 0.25 and Cts of *GAPDH* and *GUSB* not consistent. Outliers (identified using interquartile ranges) were removed from the analysis.

### BDNF protein detection

The remaining whole blood was centrifuged at 2000 rpm 10 minutes and the plasma obtained was frozen at −80 °C until use. BDNF was measured in duplicates using RayBio® Human BDNF ELISA Kit following supplier instructions.

### Western Blot analysis

Leukocytes were obtained from pelleted blood cells after red blood cells lysis with RBC Lysis Buffer (21205, Norgen Biotek corp) following supplier instructions. Proteins were extracted, separated by SDS-page in polyacrylamide gels, and transferred to nitrocellulose membranes (1620112, Bio-Rad). Membranes were blocked for 1 hour at room temperature, primary antibodies were incubated overnight at 4 °C and then with their corresponding secondary HRP-conjugated antibodies for 1 hour (1:5000, Invitrogen). Protein signal was detected with ECL chemiluminiscent system (Amersham, GE Healthcare) in ImageQuant LAS 500 (GE Healthcare). Images were processed with Image Studio Lite 5.2 (LI-COR). Densitometry analysis was performed using gapdh as loading control and ImageJ software (National Institutes of Health). Primary antibodies: rabbit anti- α2δ2 (1:500, Abgent, AP13380C), rabbit anti-MeCP2 (1:1500, Millipore, ABE333), rabbit anti-TSP3 (1:1000, Abgent, AP18972a), rabbit anti-LIN28A (1:200, #8641, Cell Signaling) and mouse anti-Vinculin (1:750, SCBT, sc-59803). Full length gels are shown in Supplementary info file.

### Data analysis

SPSS program (IBM SPSS Statistics 24.0) was used to analyze data.

Two tailored non-parametric Kruskal Wallis and Mann-Whitney U tests were used to establish metabolic and gene expression differences between conditions and to correlate candidate biomarkers with clinical variables respectively, as the sample size was not enough to presume normality within the data. Signification values in multiple comparisons were adjusted by Bonferroni correction to reduce Type 1 Error. A bivariate correlation test was used to correlate quantitative variables while Pearson product-moment correlation was used if both variables were qualitative.

To assess biomarker feasibility Receiver Operating Characteristic (ROC) curves and the area under the ROC curve (AUC) were performed. AUC vary from 0 to 1, where values 0.8 ≤ AUC < 0.9 reflect good and 0.9 ≤ AUC ≤ 1 very good biomarker performance. Statistical significance was set at p values *p < 0.05, **p < 0.01 and ***p < 0.001.

## Supplementary information

Dataset 1
