# Supplementary material for: Discovery of Biomarker Panels for Neural Dysfunction in Inborn Errors of Amino Acid Metabolism
Source: Sci Rep. 2019 Jun 24;9:9128. doi: 10.1038/s41598-019-45674-2 (PMC6591213; doi:10.1038/s41598-019-45674-2)

Supplementary files:

DISCOVERY OF BIOMARKER PANELS FOR NEURAL DYSFUNCTION IN INBORN ERRORS OF AMINO ACID METABOLISM

Alba-Aina Castells (*)[1,2], Daniela Gueraldi[1] (*), Rafel Balada[2], Alba Tristán-Noguero[1], Elisenda Cortès-Saladelafont[1], Federico Ramos[1], Silvia Meavilla[1], De los Santos M[1], Camila Garcia-Volpe[1], Roser Colomé[1], Maria Luz Couce[3], Cristina Sierra[1], Aida Ormazábal[1], Marta Batllori[1], Rafael Artuch[1], Judith Armstrong[1], Soledad Alcántara (**)[2], Àngels Garcia-Cazorla[1] (**).

Full length gels corresponding to the Figure 1d:

α2δ2 (1:500, Abgent, AP13380C)

MW Marker

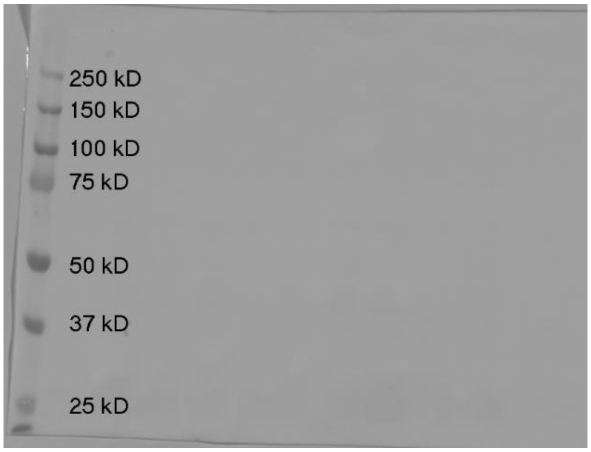

Exposure

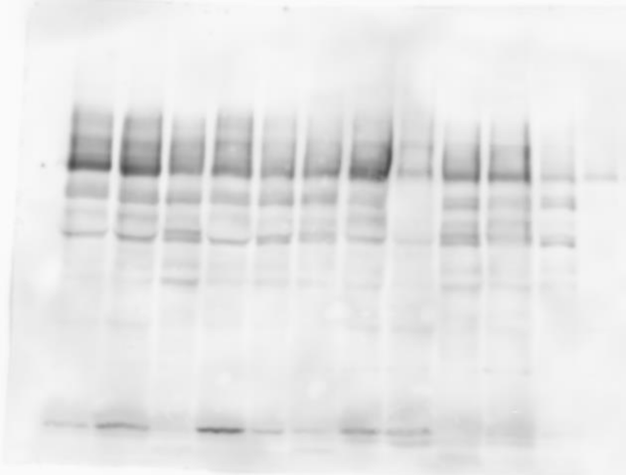

Vinculin α2δ2 gel (1:750, SCBT, sc-59803)

MW Marker

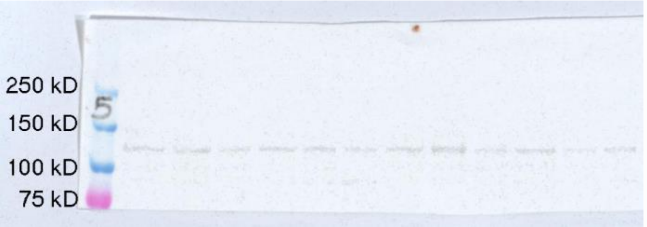

Exposure

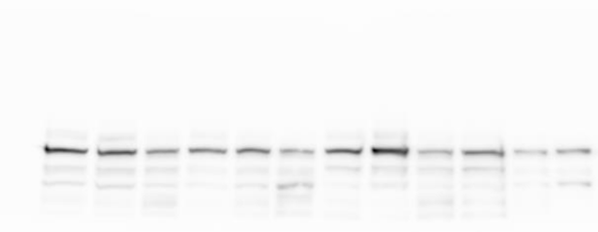

MeCP2 (1:1500, Millipore, ABE333)

MW Marker

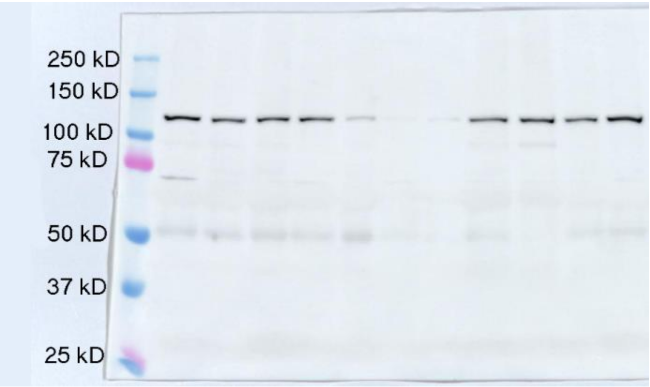

Exposure

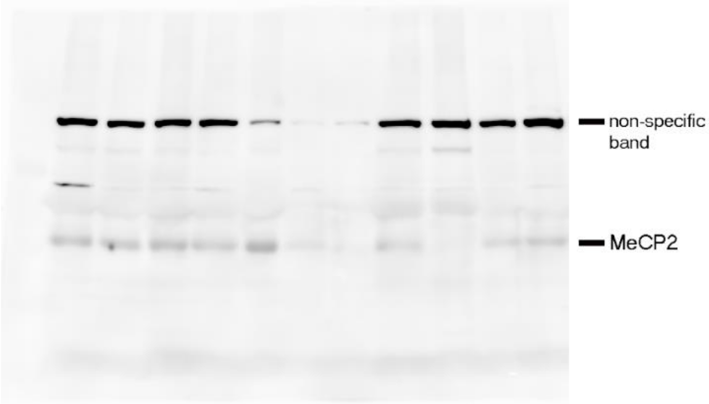

Vinculin MeCP2 gel (1:750, SCBT, sc-59803)

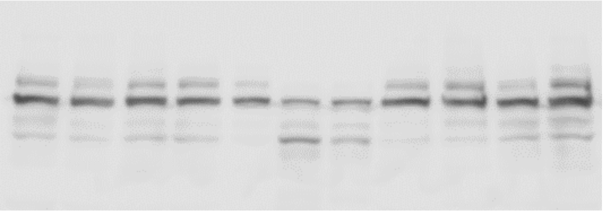

Supplement: Supplementary file 1 — Dataset 1 [file 41598_2019_45674_MOESM1_ESM.pdf]
